# Serum insulin‐like growth factor‐1 concentrations in healthy cats before and after weight gain and weight loss

**DOI:** 10.1111/jvim.16119

**Published:** 2021-04-08

**Authors:** Eric Zini, Elena Salesov, Anke Willing, Carlo Palizzotto, Thomas A. Lutz, Claudia E. Reusch

**Affiliations:** ^1^ Clinic for Small Animal Internal Medicine, Vetsuisse Faculty University of Zurich Zurich Switzerland; ^2^ Department of Animal Medicine, Production and Health University of Padova Legnaro (PD) Italy; ^3^ AniCura Istituto Veterinario Novara Granozzo con Monticello (NO) Italy; ^4^ Institute of Veterinary Physiology, Vetsuisse Faculty, University of Zurich Zurich Switzerland

**Keywords:** acromegaly, diabetes mellitus, feline, obesity

## Abstract

**Background:**

Measurement of serum concentrations of insulin‐like growth factor (IGF)‐1 is used to diagnose acromegaly in cats.

**Hypothesis:**

Changes of body weight do not affect serum concentrations of IGF‐1 in cats.

**Animals:**

Ten healthy purpose‐bred cats.

**Methods:**

Prospective study. In lean cats, food availability was stepwise increased during the first week and given ad libitum for a total of 40 weeks to increase their body weight. From week 41 to week 60, food access was limited to reach a weight loss of 1% to 2% each week. Measurement of IGF‐1 was performed at week 0, 16, 40, and 60. Insulin‐like growth factor‐1 was measured by radioimmunoassay. Body weight and IGF‐1 were compared among the 4 time points.

**Results:**

Body weight increased by 44% from week 0 (4.5 ± 0.4 kg) to week 40 (6.5 ± 1.2 kg) (*P* < .001) and decreased by 25% from week 40 to week 60 (4.9 ± 0.7 kg) (*P* < .001). Serum IGF‐1 concentrations did not differ during the study period (week 0, 16, 40, 60: 500 ± 188, 479 ± 247, 470 ± 184, 435 ± 154 ng/mL, respectively; *P* = .38). Correlations with body weight were not observed.

**Conclusions and Clinical Importance:**

Insulin‐like growth factor‐1 might not be influenced by changes of body weight in healthy cats, possibly suggesting that the latter is unimportant when interpreting IGF‐1 results in this species.

**Abbreviations**
IGF‐1: insulin‐like growth factor‐1
IGFBPs: IGF‐1 binding proteins

## 1 INTRODUCTION

Insulin like growth factor‐1 (IGF‐1) is a single chain polypeptide which acts in synergy with growth hormone (GH) to mediate its anabolic effects.^1^, ^2^ Insulin‐like growth factor‐1 is mostly secreted by the liver under GH induction.^3^ Insulin, through an upregulation of hepatic GH receptors, increases IGF‐1 secretion.^4^ Serum IGF‐1 concentration reflects GH activity during the 24 hours and its measurement is employed as diagnostic test to assess GH disorders in humans.^5^, ^6^ Circulating IGF‐1 consists of free IGF‐1, the active form of the hormone, and IGF‐1 bound to specific proteins (ie, IGF‐1 binding proteins [IGFBPs]).^7^ Obese people have lower circulating IGFBPs leading to increased availability of free IGF‐1, which causes a negative feedback on GH; the drop of GH decreases IGF‐1 release.^8^, ^9^

In cats, IGF‐1 measurement is used to diagnose acromegaly, a disease characterized by aberrant GH production because of pituitary adenoma.^10^ Other than acromegaly different conditions influence IGF‐1 activity in cats. Reduced concentrations of circulating IGF‐1 occur in newly diagnosed diabetic cats before the beginning of treatment because of the lack of insulin upregulation of hepatic GH receptors.^11^ Decreased IGF‐1 concentrations in cats also occur with lymphoma, hyperthyroidism and with aging.^11^, ^12^, ^13^ Information about the effects of body weight change on circulating IGF‐1 levels is conflicting. A lack of correlation between body weight and IGF‐1 is reported in healthy and in diabetic cats,^11^ while other studies report a positive correlation in healthy cats and in cats with different diseases^13^; IGF‐1 increases by 38% for each kilogram increase in body weight. Furthermore, after a 42% daily energy intake restriction, serum IGF‐1 and body weight significantly decreased in non‐obese cats, whereas IGF‐1 concentrations increase in obese cats after weight loss.^14^, ^15^ Insulin‐like growth factor‐1 binding proteins can interfere with IGF‐1 measurement resulting in falsely increased or decreased concentrations, depending on the assay.^12^, ^13^

In light of the conflicting results reported for IGF‐1 concentrations in relation to body weight, the aim of this prospective study was to evaluate the effect of body weight changes imposed by overfeeding and food restriction, respectively, on circulating IGF‐1 in healthy cats.

## 2 MATERIALS AND METHODS

### 2.1 Animals

The study protocol was approved by the Veterinary Office of the Canton of Zurich and conducted in accordance with guidelines established by the Animal Welfare Act of Switzerland (permission number: 118/16). Ten healthy purpose‐bred male castrated domestic short hair cats were used for the study. Median age was 6.5 years (range, 6.3‐6.6). Initially, median body weight was 4.5 kg (range, 3.6‐5.1) and body condition score, as assessed on a 9‐point scale,^16^ was 5 for all cats. The cats were group housed in the animal research facility of the Vetsuisse Faculty, University of Zurich (Switzerland).

### 2.2 Feeding plan

The study period was 60 weeks. Throughout the investigation, the 10 cats were fed a commercial dry food (Science Diet Adult Optimal Care, Hill's Pet Nutrition, Topeka, Kansas). General conditions were monitored on a daily basis and body weight and physical examination were assessed weekly in all cats. During the first week each cat was fed separately. To avoid vomiting because of overeating the daily ration was increased stepwise. In particular, during the first 2 days each cat was fed the resting energy requirement (RER)^17^ increased by 50%. The daily RER was doubled on day 3 and 4. On day 5 and 6, the daily ration was increased to 2.5‐fold of the RER. From day 7, if cats did not vomit, they were housed in a single group and the food was provided ad libitum until week 40. Body condition score was noted at the end of week 40.

From week 41, the cats were fed separately and the amount of food was reduced stepwise to reach the daily RER, mirroring the approach used during the first week. Thereafter, body weight was assessed every week and food access was individually limited to achieve a weight loss of around 1% to 2% each week, until week 60.

Body weights were measured in week 0, 16, 40, and 60.

### 2.3 Sampling

At week 0, 16, 40, and 60, cats underwent a physical examination and were sedated with 0.1 mg/kg of midazolam (Dormicum, Roche AG, Basel, Switzerland) and 10 mg/kg of ketamine (Keta‐S, Graeub, Bern, Switzerland) for blood sampling. Blood was collected from the jugular vein. Samples for cell blood counts and biochemical profiles were analyzed by standard methods in the clinical laboratory of the faculty. Serum samples were stored at −80°C to determine IGF‐1 concentrations in batch.

### 2.4 Insulin‐like growth factor‐1 measurement

Serum IGF‐1 was measured by radioimmunoassay (Mediagnost IGF‐R20, Mediagnost, Reutlingen, Germany) at the NationWide Specialist Laboratories, Pampisford, Cambridge, UK. Briefly, 10 μL of cat serum was diluted in 1 mL of acidic buffer solution (pH <3.1) to dissociate IGF‐1 from IGFBPs. Then, an IGF‐1 anti‐rabbit polyclonal first antibody with IGF‐2 excess was diluted in a buffer able to neutralize the acidic sample (pH 7). The IGF‐2 excess binds to IGFBPs while the free IGF‐1 is then measurable; with this procedure IGFBPs interference was neutralized by IGF‐2. Thereafter, the sample was further processed using radioimmunoassay and a second anti‐rabbit polyclonal antibody for IGF‐1. Sensitivity of radioimmunoassay for IGF‐1 was 0.02 to 0.109 μg/L and no cross reactivity with IGF‐2 was documented (ie, 0.1%). Intra‐assay variability was 4.8% (range, 1.0%‐16.1%) and inter‐assay variability was 5.1% (range, 4.5%‐6.0%), as reported by the laboratory. Further assay validation was achieved in cats by other authors.^18^

### 2.5 Statistical analysis

Data were tested for Gaussian distribution with Shapiro‐Wilk normality test; if non‐normally distributed, they were log‐transformed. Time points were compared for body weight and IGF‐1. Comparisons were performed with 1‐way analysis of variance for repeated measures followed by Tukey's multiple comparisons test. Each *P*‐value was adjusted to account for multiple comparisons. In addition, at each time point, correlations between body weight and IGF‐1 were assessed with Pearson correlation coefficients. Commercial software was used for statistical analysis (GraphPad Prism 7.0, San Diego, California).

## 3 RESULTS

**FIGURE 1:**
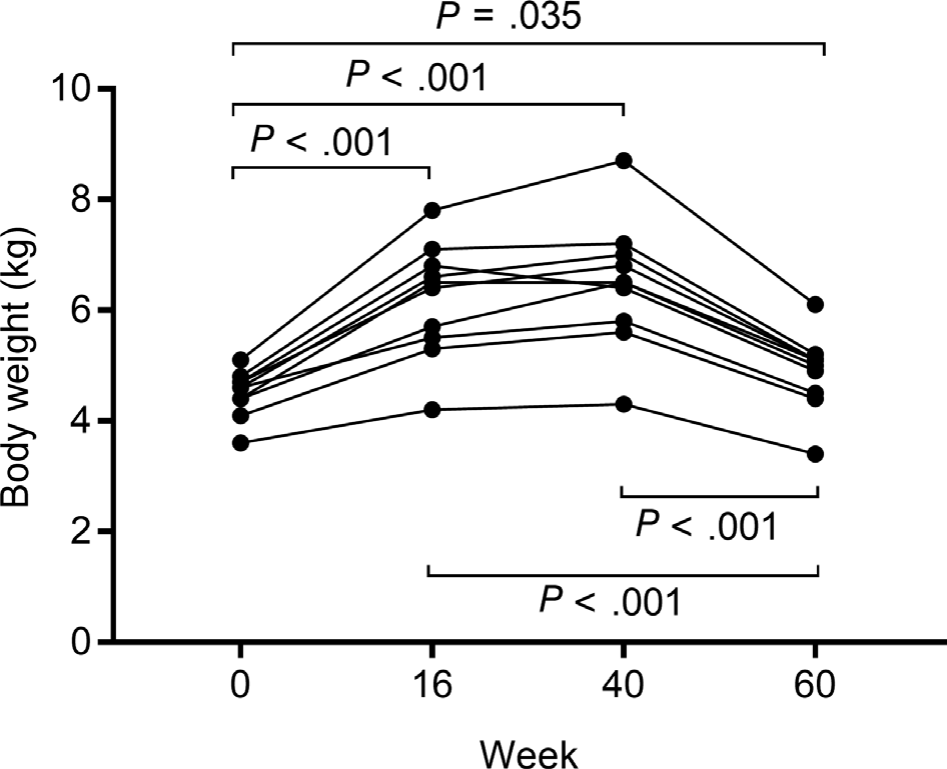
Before and after dot plot of body weight in the 10 healthy cats during the study period. A solid line connects values recorded in the same cat. Significant differences between time points are reported

**FIGURE 2:**
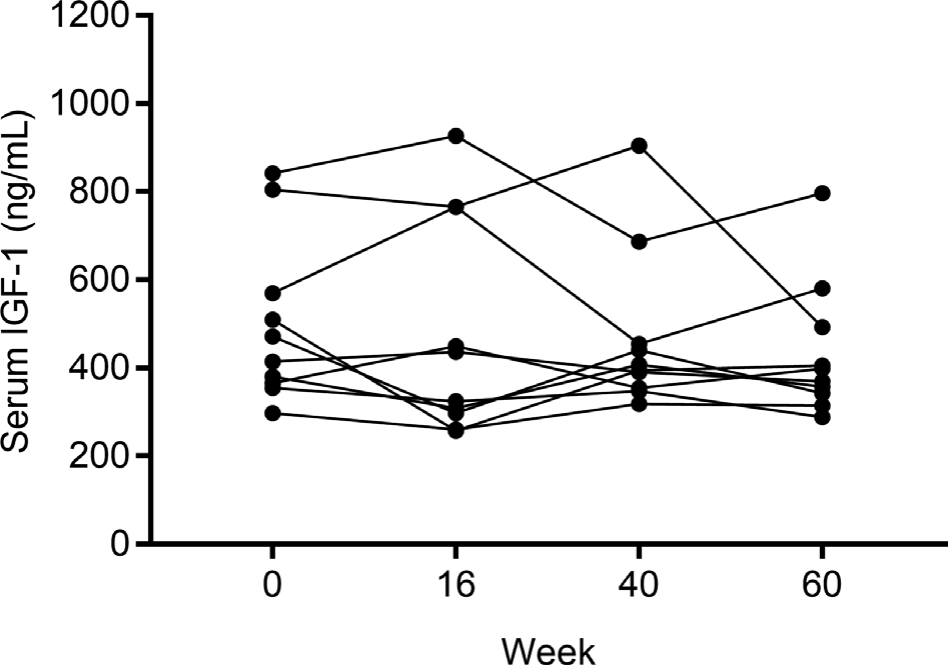
Before and after dot plot of serum IGF‐1 concentrations in the 10 healthy cats during the study period. A solid line connects values recorded in the same cat. No significant differences were observed between time points

During the study period, no cat had diarrhea but 7 of the 10 cats vomited on 1 to 2 episodes. Vomiting was assumed to be because of fast eating and overeating and in all cases was self‐limiting; medical treatment was not necessary. At week 40, 1 cat reached a body condition score of 7/9, 4 cats 8/9 and 5 cats 9/9. Body weight and IGF‐1 concentrations at each time point are reported in Table 1. Body weight significantly changed during the study period (*P* < .001) (Figure 1). In particular, average body weight increased by 44% from week 0 to week 40 and decreased by 24.7% from week 40 to week 60; of note, body weight at week 60 was still higher than at week 0 by 8.4%. Serum concentrations of IGF‐1 did not differ during the study period (*P* = .38) (Figure 2); none of the IGF‐1 measurements was >1000 ng/mL, which is the threshold commonly reported for acromegaly,^18^ at any time point. Correlations between body weight and IGF‐1 were not observed (week 0: *r* = 0.531, 95% CI = −0.148 to 0.870, *P* = .11; week 16: *r* = 0.351, 95% CI = −0.357 to 0.803, *P* = .32; week 40: *r* = 0.622, 95% CI = −0.011 to 0.899, *P* = .06; week 60: *r* = 0.384, 95% CI = −0.323 to 0.816, *P* = .27).

**TABLE 1:** Mean, standard deviation, and range of body weight and of serum concentrations of IGF‐1, in the 10 healthy cats during the study period

	Week 0	Week 16	Week 40	Week 60
Body weight (kg)	4.5 ± 0.4 (3.6‐5.1)	6.2 ± 1.3 (4.2‐7.8)	6.5 ± 1.2 (4.3‐8.7)	4.9 ± 0.7 (3.4‐6.1)
IGF‐1 (ng/mL)	500 ± 188 (297‐841)	479 ± 247 (257‐926)	470 ± 184 (318‐904)	435 ± 154 (288‐796)

Among blood work variables, during the study serum cholesterol and triglycerides concentrations were increased above the reference interval in 5 cats and 1, respectively. The remaining of the biochemical profile and cell blood counts was within the reference interval.

## 4 DISCUSSION

The present study shows that an increase of body weight followed by its partial reduction did not affect serum IGF‐1 concentrations in healthy cats. In addition, there was no correlation between body weight and IGF‐1 before and after weight gain. These results confirm a previous study where correlations between body weight and serum IGF‐1 concentrations were not documented in 18 healthy cats.^11^ Nevertheless, contrasting results have been reported between body weight and serum IGF‐1 in cats.^13^, ^14^, ^15^ In particular, in a recent investigation assessing circulating IGF‐1 in 55 healthy cats and in 10 cats with a variety of diseases, a positive correlation with body weight was observed; in the same study no longitudinal assessment of IGF‐1 and body weight was performed to provide more information about their relationship.^13^ In humans no correlation was identified between IGF‐1 concentrations and body weight, while IGF‐1 was negatively correlated to body mass index, a parameter used to assess the nutritional status.^19^, ^20^ It was assumed that obesity lowers circulating IGFBPs, leading to a decrease of IGF‐1.^8^, ^9^ Unfortunately, details regarding the nutritional status of the cats enrolled in the previous studies were scant to gain further insights.

In cats, body condition score is used to assess the nutritional status and reliably semiquantifies the body fat percent.^16^, ^21^ In the present series, the 10 cats had a score of 5/9 at the study beginning which increased to at least 7/9 at the end of the ad libitum period in all, while IGF‐1 concentrations did not change, suggesting that IGF‐1 is not influenced by augmenting body fat in this species.

In the present cats, food availability was gradually reduced from week 41 to week 60 to achieve weight loss. Despite the significant decrease in body weight, no change in IGF‐1 concentrations was observed. A previous study evaluated the effects of weight loss in obese cats and it was reported that weight loss was associated to an increase of IGF‐1 compared to baseline.^15^ Because obese cats have insulin resistance and because of the permissive role of insulin on IGF‐1 release, the authors assumed that improved insulin sensitivity after weight loss explained the increase in IGF‐1.^15^ Despite body weight reduction, our cats at week 60 were still heavier compared to week 0. Hence, we cannot exclude that insulin resistance was still present impeding an increase of IGF‐1. Another possible explanation for the difference is represented by the diet used in the studies; in particular, the present cats received a maintenance diet while in the former study a high‐protein diet was given.^15^ A study in pigs reported that high‐protein diet increased hepatic IGF‐1 gene expression with consequent increase in circulating IGF‐1.^22^ Conversely, in humans low‐protein diet increased serum IGFBPs, which in turn decreased IGF‐1 concentrations.^23^ Hence, further studies are needed to assess the relationship between diet proteins and circulating IGF‐1 in cats.

Of note, in 1 study assessing the effect of restricting the daily energy ration of healthy lean cats to 56% and to 42% during 2 weeks, it was found that body weight decreased in both cases whereas IGF‐1 concentrations decreased only in the latter.^14^ Similar to cats, different studies in humans reported that weight loss is associated with IGF‐1 reduction only when energy intake is below 50% of the daily ration.^24^, ^25^, ^26^, ^27^, ^28^ Therefore, we hypothesize that energy restriction to <50% daily ration more than body weight variation affects IGF‐1 concentrations in cats.

The study has some limitations, particularly the fact that IGF‐1 concentrations were measured without taking into account the potential bias of IGFBPs. In addition, it cannot be excluded that IGF‐1 concentrations would have required longer to increase or decrease after changes in body weight, despite the study design spanned over several months. A positive correlation between IGF‐1 and body weight was not observed at any time point. It is possible that with a larger number of cats and wider range of body weights for each body condition score, positive correlations would have been identified. Of note, with a statistical power of 0.8 the sample size of 10 cats reached significance with a correlation coefficient of 0.800. Despite correlation coefficients are usually lower in clinical studies, the present investigation was based on an obesity model in healthy cats allowing to limit the confounders and, possibly, increasing the chance of a high correlation coefficient.

Insulin‐like growth factor‐1 measurements were relatively high in 3 cats (ie, >800 ng/mL), which might be suspicious of acromegaly.^18^ One of them had concentrations >800 ng/mL in 3 of the 4 measurements, while 2 only once. Whether this suggests an early stage of acromegaly, in particular in the former cat, is unclear. Nonetheless, after 2 years from the experiment, the 3 cats were still in good health and none showed clinical signs associated with acromegaly.

In conclusion, circulating IGF‐1 might not be influenced by body weight increase and by its partial decrease in healthy cats, possibly suggesting that body weight is not important when interpreting IGF‐1 results in this species.

## CONFLICT OF INTEREST DECLARATION

Eric Zini serves as Associate Editor for the Journal of Veterinary Internal Medicine. He was not involved in the review of this manuscript. None of the authors has any financial or personal relationships that could inappropriately influence or bias the content of the paper.

## OFF‐LABEL ANTIMICROBIAL DECLARATION

Authors declare no off‐label use of antimicrobials.

## INSTITUTIONAL ANIMAL CARE AND USE COMMITTEE (IACUC) OR OTHER APPROVAL

Approved by the Veterinary Office of the Canton of Zurich and conducted in accordance with guidelines established by the Animal Welfare Act of Switzerland (permission 118/2016).

## HUMAN ETHICS APPROVAL DECLARATION

Authors declare human ethics approval was not needed for this study.
